# Antitrypanosomal potential of *Salvia officinalis* terpenoids-rich fraction in *Trypanosoma evansi*-infected rat model

**DOI:** 10.1186/s12917-025-04861-2

**Published:** 2025-06-27

**Authors:** Marian G. Sawerus, Hamdy H. Kamel, Walaa M. S. Ahmed, Emad B. Ata, Dalia El Amir, Emad A. Mahdi, Marwa A. Ibrahim, Olfat Shehata

**Affiliations:** 1 Department of Clinical Pathology, Faculty of Veterinary Medicine, Beni-Suef University, Beni-Suef, 62511 Egypt; 2 Department of Parasitology and Animal Diseases, Veterinary Research Institute, National Research Centre, Giza, 12622 Egypt; 3 Department of Pharmacognosy, Faculty of Pharmacy, Beni-Suef University, Beni-Suef, 62514 Egypt; 4 Department of Pathology, Faculty of Veterinary Medicine, Beni-Suef University, Beni-Suef, 62511 Egypt; 5 Department of Biochemistry and Molecular Biology, Faculty of Veterinary Medicine, Cairo University, Giza, 12211 Egypt

**Keywords:** *Salvia officinalis*, Terpenoids, *Trypanosome evansi*, Antitrypanosomal activity

## Abstract

**Background *Trypanosoma evansi*:**

(*T. evansi*) is a major protozoan disease that affects animals, including camels, and causes substantial economic detriments. The failure to control *T*. *evansi* infections is due to the unavailability of vaccines and the development of resistance to existing chemical drugs. In this study, we evaluated the effect of *Salvia officinalis* terpenoids-rich fraction on the degree of parasitemia and associated pathological alterations in rats experimentally infected with *T. evansi*.

**Method:**

Eighty adult male rats were equally divided into 4 groups. The first group was a negative control. The second group was intraperitoneally infected with *T. evansi* at a dose of 1 × 10^4^ trypanosomes. The third group was similarly infected and subsequently treated intramuscularly with diminazene aceturate at a dose of 7 mg/kg body weight (b.wt.). The fourth group received a daily oral administration of *Salvia officinalis* terpenoids-rich fraction at a dose of 300 mg/kg b.wt. throughout the experimental period and was also infected with *T. evansi*.

**Result:**

The infection with *T. evansi* resulted in normocytic normochromic anemia, leukocytosis, hypoglycemia, hypertriglyceridemia, an increase in very low-density lipoprotein (VLDL) cholesterol, and reductions in high-density lipoprotein (HDL) and low-density lipoprotein (LDL) cholesterols. Additionally, the infection induced upregulation of the pro-inflammatory cytokines such as interleukin-1beta (IL-1β) and interleukin-6 (IL-6), and downregulation of the anti-inflammatory cytokines such as interleukin-10 (IL-10) and transforming growth factor beta (TGF-β). Besides histopathological changes in the brain and spleen, *T. evansi* markedly elevated brain oxidative stress and acetylcholinesterase (AChE) activity. The treatment with salvia fraction significantly decreased the degree of parasitemia and mitigated the *T. evansi*–induced pathological alterations.

**Conclusion:**

The terpenoids-rich fraction from *Salvia officinalis* exhibits antitrypanosomal activity and may serve as a promising candidate for developing novel trypanocidal agents.

**Supplementary Information:**

The online version contains supplementary material available at 10.1186/s12917-025-04861-2.

## Introduction

Trypanosomiasis, a disease caused by *Trypanosoma* sp., affects both domestic animals and wildlife and is of great zoonotic importance, particularly in tropical and subtropical regions. *T. evansi*, a salivarian trypanosome transmitted mechanically through biting flies (mostly Tabanids and Stomoxes), serves as the principal causative agent of trypanosomiasis in camels, resulting in a disease called Surra (meaning emaciated or rotten) [1, 2]. This protozoon has the broadest host range with severe infections commonly observed in camels, equines, cattle, and buffaloes [3, 4]. *T. evansi* is one of the most important protozoal diseases in camels, causing a significant economic impact in the endemic areas in the form of reduced meat, milk, and work capacity in camel-rearing areas. Based on global epidemiological data, the parasite is prevalent in most countries of the Middle East with a worldwide distribution [1].

The typical clinical manifestations of the disease are pyrexia (closely related to parasitemia), anemia, weight loss, and intermittent fever due to recurrent episodes of parasitemia during the course of the disease. Urticarial plaques, petechial hemorrhages in the mucous membranes, rough coats, and edema, especially affecting the lower body, are commonly observed. Furthermore, immunosuppression is often evidenced by vaccination failures [1, 3]. The antigenic diversity of trypanosomes is considered an important adaptive mechanism that allows evasion of the host immune system, rendering the development of an effective vaccine in the future unlikely. Consequently, chemotherapy remains the predominant method to control trypanosomiasis [5]. The majority of chemotherapeutics used in the treatment of *T. evansi* infections display limited efficacy often resulting in relapsing parasitemia and associated clinical symptoms. Currently, the treatment of Surra relies on five medications: quinapyramine, melarsomine, suramin, isometamidium chloride, and diminazene aceturate, which are expensive and not universally accessible. The emergence of resistance to these medications threatens their efficacy and underscores the necessity for new therapeutic agents [6].

Since ancient times, plant products have been used to treat different illnesses and are considered a rich source of novel antiparasitic compounds [7]. Different *Salvia* species have been reported to be among the promising plant-based treatments for parasitic infestations [8]. *Salvia officinalis (S. officinalis)*, also known as common sage, is a herbal plant belonging to the Lamiaceae family. It has been utilized as a traditional treatment for various diseases, including hyperglycemia, diarrhea, dizziness, ulcers, gout, paralysis, and rheumatism [9]. Phytochemical analysis of *S. officinalis* leaf extracts reveals that they mainly comprise of mono- and diterpenes, which have been known to exhibit antibacterial, antiprotozoal, antifungal, anti-inflammatory, antioxidant, and anti-mutagenic activities [9–11]. Few studies have proved the efficacy of isolated compounds from those plants against protozoan parasites such as *Trypanosoma*, *Leishmania*, and *Plasmodium* [8, 12]. In vitro, the constituents identified from *S. officinalis* and exhibiting significant activity against *Trypanosoma brucei rhodesiense* are eight diterpenes and one flavonoid [13].

Therefore, this study aimed to evaluate the trypanocidal efficacy of *S. officinalis* terpenoids-rich fraction and subsequent clinical outcome in a rat model experimentally infected with *T. evansi*. As far as we know, this will be the first report investigating the in vivo effect of *S. officinalis* terpenoids-rich fraction against *T. evansi*. The data obtained will help in developing a new approach for controlling trypanosomiasis and other blood parasites in humans and animals.

## Materials and methods

### Chemicals

Chemicals for measuring the oxidative stress biomarkers and AChE activity were purchased from Loba-chemie Co. (Mumbai, Maharashtra, India) and Carl Roth-Germany. Diminazene aceturate (BATRYNIL) was bought from Arab Company for Medical Products, Egypt.

The blood hemoglobin (Hb) and serum glucose were estimated using commercially available kits from Vitro Scient Company (Cairo, Egypt), while kits from EGY CHEM for lab technology (Badr City, Industrial Area, Cairo, Egypt) were used for lipid profile measurement. The Qiagen RNeasy kit (Qiagen AB, Hilden, Germany) was used for total RNA extraction from blood specimens. The RevertAid First Strand cDNA Synthesis Kit (Thermo Fisher Scientific Corporation, USA) was used to convert the isolated total RNA to complementary DNA (cDNA). The kit of SsoAdvanced™ Universal SYBR^®^ Green Supermix (Bio-Rad, Hercules, CA, USA) was utilized for quantitative real-time PCR (qPCR) for gene expression levels.

### Plant material

*S. officinalis* leaves were bought from Harraz^®^ market. The extraction, fractionation, and liquid chromatography–mass spectrometry (LC–MS) analysis of *S. officinalis* terpenoids-rich fraction were conducted as a part of our previous study [14] and the previously prepared extract was utilized in the current study. The powdered leaves (1.5 kg) were extracted using 80% ethanol till exhaustion using sonication three times for a duration of 20 min each. The final dry residue (157 g) of the ethanolic extract was obtained via concentration under vacuum. The residue was then suspended in distilled water (500 ml) and fractionated using ethyl acetate till exhaustion, resulting in 80 g of terpenoids-rich fraction. For LC-MS analysis, 1 g of this fraction was kept in a tightly sealed container. The fraction was dissolved in 2% Tween 80.

### *Trypanosoma evansi* isolate

The strain of *T. evansi* was isolated from naturally infected camels in the Halaib and Shalatien area on the Northeast African coast of the Red Sea in the summer of 2021. It was cryopreserved in liquid nitrogen under laboratory conditions. Depending on the pilot study, this isolate induces an acute infection in rats with a survival period of 13–15 days after infection, if not treated. Initially, two rats were intraperitoneally infected with cryopreserved blood as a means to obtain a large number of parasites for subsequent infection of the experimental groups in the current study. They were housed in a plastic cage on a day/night cycle of 12 h under controlled temperature and humidity conditions (25 ± 2 ºC; 60–70% respectively), providing water and feed *adlibitum*. They were observed daily for parasitemia, and when they became parasitemic, the number of trypanosomes in the blood was estimated using a hemocytometer to calculate the infection dose.

### Experimental design

Eighty adult male albino rats weighing 180–200 g were obtained from the Nile Company (El Amyria, Cairo, Egypt) and housed in plastic cages, five rats each, under controlled environmental and light conditions with free access to water and feed as previously described in section “*Trypanosoma evansi* isolate”. All animals were subjected to a ten-day acclimatization period for adaptation to the laboratory environment.

The rats were divided equally into four groups. The negative control (CN) group was non-infected and non-treated. The positive control (CP) group was infected intraperitoneally with 1 × 10^4^ trypanosomes [15]. The DA group was infected and treated with a standard chemical drug (diminazene aceturate) as a single intramuscular dose of 7 mg/kg b.wt. [16]. when all infected rats became parasitemic (on the 8^th^ day post-infection). The salvia fraction (SF) group was treated with *S. officinalis* terpenoids-rich fraction at 300 mg/kg b.wt. and infected. The fraction was administered orally in a fixed volume of 1 mL/rat daily for 1 month (the treatment regimen commenced 2 weeks before infection and 2 weeks after infection till the end of the experiment). The other experimental groups orally received saline (1mL/rat/daily) throughout the experimental period.

The dose of salvia fraction was estimated according to the preliminary acute safety study following the Economic Co-operation and Development (OECD) guideline number 423 [14]. The 300 mg/kg b.wt. of salvia fraction (1/10 of 3000 mg/kg b.wt.) was selected as the maximum safety dose that can be administered daily over a prolonged period.

### Parasitemia estimation

A preliminary blood examination was done daily after animal infection to assess the presence of trypanosomes. It was performed through wet preparation using a drop of blood from the tail vein placed on a clean glass slide, covered with a glass cover, and examined under the microscope. After the parasite detection in the blood, the parasitemia degree was estimated using a Neubauer hemocytometer according to the method previously reported by Dyary et al. [17].

### Sample collection

Five rats from each group were randomly selected and subjected to deep anesthesia using isoflurane for blood collection on day zero (before infection), as well as on the 10^th^ and 14^th^ day post-infection (DPI). A part of the blood samples was collected in sample tubes containing ethylenediaminetetraacetic acid (EDTA), an anticoagulant, for hematological parameters and gene expression for IL-1β, IL-6, IL-10, and TGF-β. For biochemical tests, the other part was collected into plain tubes for serum separation. The serum samples were then preserved at -20℃ until analysis.

On the 14^th^ DPI, following blood collection, the rats were humanely euthanized, and the organs, including the brain and spleen, were collected. The brains were washed with saline and divided into two portions; the first portion was homogenized for oxidative stress biomarkers and AChE activity estimations. The second portion of brain tissue, along with the spleen, was preserved in a 10% formalin solution for histopathological examination. After fixation in 10% formalin solution, they were washed in water and dehydrated in ascending grades of alcohol, then cleared in xylene. Samples were embedded in paraffin to prepare four µm paraffin sections and stained with hematoxylin and eosin stain (H&E) as described by Bancroft and Layton [18].

### Hematology

The total red blood cell (RBC) and white blood cell (WBC) counts were accomplished by haemocytometer method [19]. The packed cell volume (PCV) was determined in accordance with Thrall et al. [20]. The blood hemoglobin (Hb) estimation was measured colorimetrically, as described by Drabkin and Austin [21]. Calculation of blood indices, including mean corpuscular volume (MCV), mean corpuscular hemoglobin (MCH), and mean corpuscular hemoglobin concentration (MCHC) were conducted as previously mentioned by Dacie and Lewis [22] A differential WBC count was conducted and calculated according to Jain [23].

### Clinical biochemistry

Serum glucose level, total cholesterol, high-density lipoprotein (HDL) cholesterol, and triglycerides were measured by spectrophotometer using commercially available kits, following the recommended protocol of the manufacturer. The estimation of low-density lipoprotein (LDL) and very low-density lipoprotein (VLDL) cholesterol concentrations was conducted employing Friedewald’s formula (VLDL cholesterol = Triglycerides/5, and LDL cholesterol = Total cholesterol - (VLDL + HDL cholesterol)).

### Brain oxidative stress biomarkers and AChE activity

The brain tissue was homogenized at a concentration of 10% w/v in 1x phosphate buffer saline (pH 7.4), followed by centrifugation in K3 series benchtop centrifuges at 3000 r.p.m for 15 min at 4 °C. The supernatant was separated and used for oxidative stress biomarkers and AChE activity estimations.

The malondialdehyde (MDA) concentration in brain homogenate was measured calorimetrically using thiobarbituric acid (TBA), following the procedure outlined by Albro et al. [24], and the concentration was expressed as nmol/100 mg tissue. The estimation of reduced glutathione (GSH) was conducted using Ellman reagent (5,5′-dithiobis-(2-nitrobenzoic acid) or DTNB) as described by Sedlak and Lindsay [25], and the result was expressed as nmol/100 mg tissue. The glutathione peroxidase (GPx) activity was estimated in accordance with Rotruck et al. [26], and the activity was expressed as nmol GSH consumed/min/100 mg tissue. The AChE activity was assessed using the DTNB reagent, following the methodology of Ellman et al. [27] with the modification reported by Gorun et al. [28]. The result was expressed as nmol thiocholine released/min/100 mg tissue at 37 ℃.

### Gene expression levels of inflammatory cytokines

Total RNA was extracted from blood samples using the Qiagen RNeasy kit, following the manufacturer’s guidelines. The purity and concentration of the isolated tRNA were measured spectrophotometrically. Conversion of the isolated RNA to cDNA was done using RevertAid First Strand cDNA Synthesis Kit, and the quantification of IL-1β, IL-6, IL-10, and TGF-β gene expression levels was performed by a real-time PCR system (Applied Biosystems, USA) using SsoAdvanced™ Universal SYBR^®^ Green Supermix in accordance with the manufacturer’s instructions. The beta-actin gene (ACTB) was used as a control endogenous housekeeping gene to normalize gene expression data using the f0 method [29]. The primer sets used in the study are shown in Table 1.

**Table 1:** Sequence of primers used in quantitative PCR (qPCR)

Gene	Forward primer (5′ -3ˊ)	Reverse primer (5ˊ-3ˊ)	Amplicon size	Accession number
IL-1β	TTGAGTCTGCACAGTTCCCC	GTCCTGGGGAAGGCATTAGG	161	NM_031512.2
IL-6	CCAGTTGCCTTCTTGGGACT	TCTGACAGTGCATCATCGCT	224	NM_012589.2
IL-10	TCCCTGGGAGAGAAGCTGAA	CCTGCAGTCCAGTAGATGCC	234	NM_012854.2
TGF-β	CACTCCCGTGGCTTCTAGTG	GGACTGGCGAGCCTTAGTTT	145	NM_021578.2
ACTB	CCGCGAGTACAACCTTCTTG	CAGTTGGTGACAATGCCGTG	297	NM_031144.3

## Statistical analysis

The data were analyzed using IBM SPSS version 26 statistical software. The results are expressed as means ± standard error (SE), and significant differences between groups were clarified through one-way analysis of variance (one-way ANOVA) followed by the Tukey post-hoc test. The differences were statistically significant at *P < 0.05*.

## Results

### LC-MS analysis of *S. officinalis* terpenoids-rich fraction

The phytoconstituents of *S. officinalis* fraction are shown in Table 2.

**Table 2:** Phytoconstituents of *S. officinalis* fraction

Class	Compounds	Relative percent %
Diterpenes	Rosmanol methyl ether isomer	18.028
	Romansol methyl ether	11.377
	Epiisorosmanol	8.635
	Carnosic acid	4.212
	Rosmanol isomer	3.818
	Carnosol	3.365
	Rosmaridiphenol isomer	0.712
	12-Methoxy carnosic acid	0.695
	Rosmaridiphenol	0.352
Triterpenes	Ursolic acid	10.076
	Micromeric acid	4.422
	Asiatic acid	3.788
Flavonoids	Rhamnetin	5.101
	Pectolinarigenin	3.121
	Apegenin-7-O-glucoside	1.266
	Cirsimaritin	0.781
	Luteolin-7-O-rutinoside	0.684
	Luteolin-7-O-glucoside	0.518
Phenolic acids	Gallic acid	15.045
	Caffeic acid hexoside	1.471
	Ferulic acid	1.321
Tannin	Catechin	1.212

As previously reported by Sawerus et al. [14]

### Parasitemia estimation

**Fig. 1:**
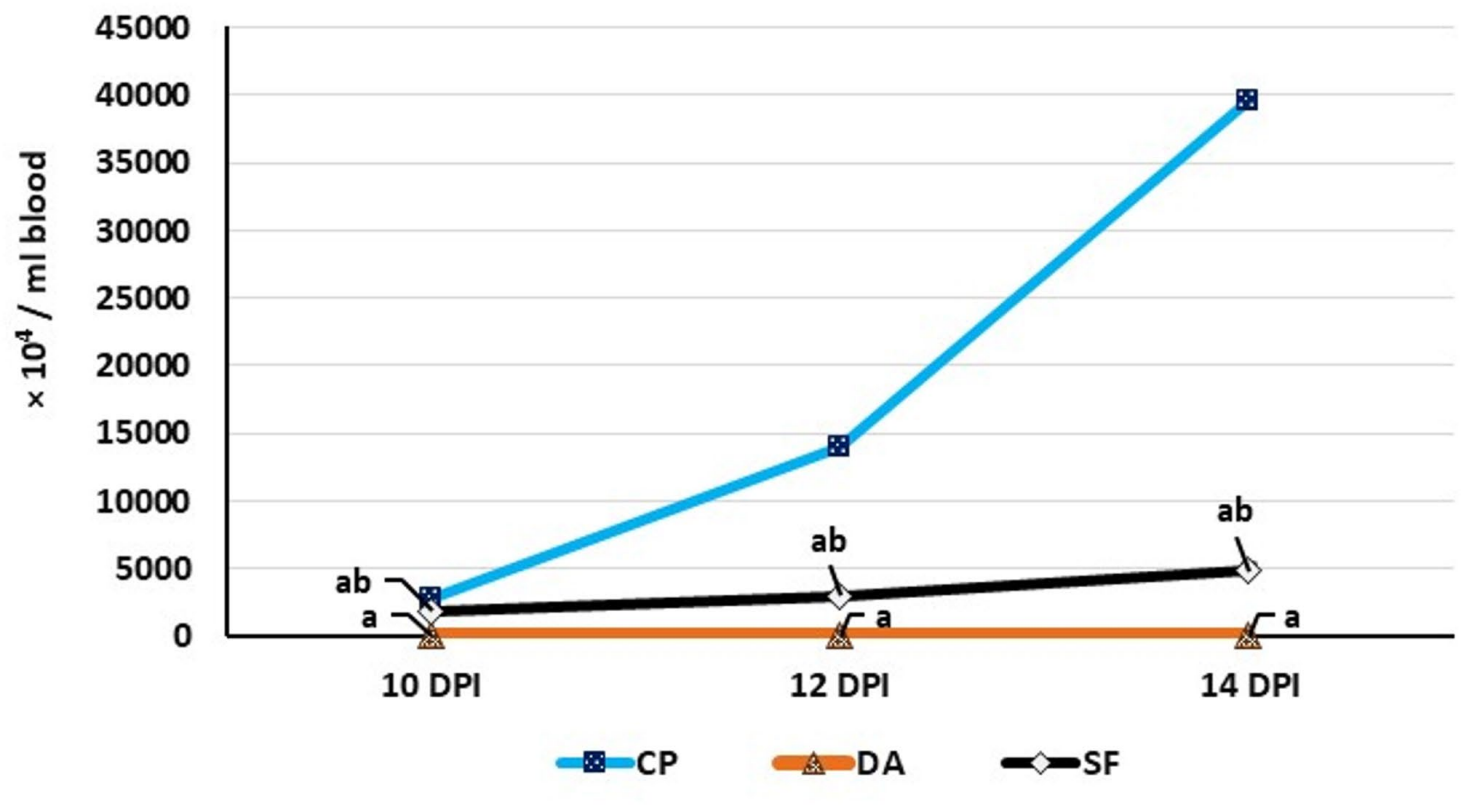
Parasitemia degree in the infected groups. Data are expressed as means ± SE (*n* = 5) with dissimilar superscript letters (significantly differing at *P < 0.05*): **a**) significantly different from CP group; **b**) significantly different from DA group. CP: positive control group (infected); DA: infected and diminazene aceturate-treated group; SF: salvia fraction-treated and infected group. DPI: day post-infection

The prepatent period was between the 7^th^ and 8^th^ DPI in all infected groups. The rats in the CP group showed progressively increasing parasitemia, while the parasite couldn’t be detected in the blood smears of rats in the DA group after diminazene aceturate administration up to the 14^th^ DPI. The oral administration of salvia fraction resulted in a significant reduction in the level of parasitemia compared to the CP group, as shown in Fig. 1.

### Hematology

On day zero, no statistically significant variations were observed among the experimental groups in the erythrogram and leucogram.

On the 10^th^ DPI, a notable reduction in the total RBC count, PCV, and Hb concentration occurred with no variation in MCV and MCHC, indicating normocytic normochromic anemia in all infected groups compared to the CN group. On the 14^th^ DPI, no statistically significant variation in blood indices was observed among all the experimental groups. A significant reduction in total RBC count, PCV, and Hb concentration was determined in the CP and SF groups in comparison to the CN group. Conversely, in treated groups (DA and SF), their values were markedly elevated compared to the CP group **(**Table 3**)**.

On the 10^th^ DPI, statistically significant leucocytosis and monocytosis were observed in all infected groups compared to the CN group. A significant lymphocytosis was observed in both the CP and SF groups, with the CP group also exhibiting neutrophilia compared to the CN group. On the other hand, a statistically significant decrease in total leucocytic count, lymphocytes, neutrophils, and monocytes was determined in both treated groups (DA and SF) compared to the CP group. On the 14^th^ DPI, relative to the CN group, a significant leucocytosis accompanied by lymphocytosis and monocytosis was observed in the CP and SF groups. The neutrophils were significantly elevated only in the CP group compared to the CN group. Leukocytosis, lymphocytosis, neutrophilia, and monocytosis, induced by the infection, were significantly decreased in the DA and SF groups compared to the CP group, with this reduction being more pronounced in the DA group than in the SF group **(**Table 4**)**.

**Table 3:** Erythrogram of the different experimental groups

Parameters Groups		Erythrocyte (×10^6^ /µL)	PCV %	Hb (g/dl)	MCV (fl)	MCH (pg)	MCHC (%)
Day zero	CN	9.51 ± 0.02	43.33 ± 0.88	11.42 ± 0.02	45.58 ± 0.92	12.02 ± 0.04	26.38 ± 0.48
	CP	9.56 ± 0.02	44.33 ± 0.67	11.24 ± 0.52	46.41 ± 0.81	11.77 ± 0.58	25.33 ± 0.82
	DA	9.29 ± 0.09	43.67 ± 0. 88	11.53 ± 0.31	47.04 ± 1.33	12.42 ± 0.38	26.41 ± 0.38
	SF	9.49 ± 0.07	43.67 ± 0.88	11.14 ± 0.33	45.99 ± 0.75	11.73 ± 0.32	25.50 ± 0.28
10^th^ DPI	CN	9.53 ± 0.34	46.33 ± 0.67	13.33 ± 0.04	48.75 ± 1.71	14.03 ± 0.52	28.79 ± 0.36
	CP	7.19 ± 0.15 ^a^	40.67 ± 0.88 ^a^	10.18 ± 0.53 ^a^	56.58 ± 0.09	14.20 ± 0.98	25.11 ± 1.76
	DA	7.57 ± 0.09 ^a^	43.00 ± 0.58 ^a^	11.51 ± 0.15 ^a^	56.78 ± 0.61	15.17 ± 0.39	26.77 ± 0.71
	SF	7.79 ± 0.21^a^	43.33 ± 0.33 ^a^	10.81 ± 0.50 ^a^	55.67 ± 1.33	13.87 ± 0.50	24.95 ± 1.25
14^th^ DPI	CN	9.67 ± 0.08	46.67 ± 0.67	13.72 ± 0.87	48.27 ± 0.39	14.20 ± 0.99	29.46 ± 2.24
	CP	6.13 ± 0.28^a^	30.67 ± 1.20 ^a^	7.84 ± 0.46 ^a^	50.21 ± 3.07	12.87 ± 1.17	25.55 ± 0.89
	DA	8.51 ± 0.44 ^b^	45.00 ± 0.58 ^b^	12.02 ± 0.46 ^b^	53.10 ± 2.35	14.26 ± 1.32	26.75 ± 1.37
	SF	7.90 ± 0.35 ^ab^	41.33 ± 0.67 ^ab^	10.39 ± 0.39 ^ab^	52.56 ± 3.01	13.19 ± 0.67	25.18 ± 1.33

Data are expressed as means ± SE (*n* = 5) with dissimilar superscript letters (significantly differing at *P < 0.05*): (a) significantly different from CN group; (b) significantly different from CP group. CN: negative control group; CP: positive control group (infected); DA: infected and diminazene aceturate-treated group; SF: salvia fraction-treated and infected group. PCV: packed cell volume; Hb: hemoglobin; MCV: mean corpuscular volume; MCH: mean corpuscular hemoglobin; MCHC: mean corpuscular hemoglobin concentration; DPI: day post-infection

**Table 4:** Leucogram of the different experimental groups

Parameters Groups		TLC (×10^3^ /µL)	Lymphocyte (×10^3^ /µL)	Neutrophil (×10^3^ /µL)	Eosinophil (×10^3^ /µL)	Monocyte (×10^3^ /µL)
Day zero	CN	10.02 ± 0.27	8.15 ± 0.30	1.14 ± 0.06	0.23 ± 0.12	0.50 ± 0.01
	CP	10.00 ± 0.23	8.07 ± 0.17	1.20 ± 0.04	0.27 ± 0.04	0.47 ± 0.07
	DA	10.32 ± 0.15	8.14 ± 0.11	1.28 ± 0.04	0.38 ± 0.09	0.52 ± 0.01
	SF	10.02 ± 0.16	8.14 ± 0.26	1.10 ± 0.16	0.34 ± 0.04	0.44 ± 0.04
10^th^ DPI	CN	8.75 ± 0.44	6.97 ± 0.35	1.17 ± 0.07	0.32 ± 0.04	0.29 ± 0.02
	CP	16.77 ± 0.27 ^a^	12.41 ± 0.47 ^a^	2.56 ± 0.36 ^a^	0.51 ± 0.18	1.28 ± 0.06 ^a^
	DA	11.55 ± 0.35 ^ab^	8.61 ± 0.36 ^b^	1.72 ± 0.08 ^b^	0.46 ± 0.01	0.76 ± 0.05 ^ab^
	SF	12.67 ± 0.86 ^ab^	9.87 ± 0.56 ^ab^	1.68 ± 0.12 ^b^	0.43 ± 0.07	0.69 ± 0.16 ^ab^
14^th^ DPI	CN	9.15 ± 0.44	7.38 ± 0.31	1.07 ± 0.02	0.25 ± 0.04	0.46 ± 0.08
	CP	18.17 ± 0.13 ^a^	14.23 ± 0.24 ^a^	2.24 ± 0.06 ^a^	0.24 ± 0.06	1.45 ± 0.10 ^a^
	DA	10.03 ± 0.64 ^b^	7.88 ± 0.45 ^b^	1.40 ± 0.13 ^b^	0.29 ± 0.00	0.48 ± 0.06 ^b^
	SF	13.20 ± 0.39 ^abc^	10.56 ± 0.33 ^abc^	1.50 ± 0.02 ^b^	0.31 ± 0.06	0.84 ± 0.09 ^abc^

Data are expressed as means ± SE (*n* = 5) with dissimilar superscript letters (significantly differing at *P < 0.05*): (a) significantly different from CN group; (b) significantly different from CP group; (c) significantly different from DA group. CN: negative control group; CP: positive control group (infected); DA: infected and diminazene aceturate-treated group; SF: salvia fraction-treated and infected group. TLC: total leucocytic count; DPI: day post-infection

### Clinical biochemistry

Regarding the glucose level and lipid profile in different experimental groups, on day zero, there was no significant variation in values of biochemical parameters among the experimental groups. However, on the 10^th^ DPI, marked hypoglycemia, accompanied by an elevation of triglycerides and VLDL cholesterol, was observed in all infected groups. In contrast, these variations were present only in the CP and SF groups on the 14^th^ DPI compared to the CN group. In comparison to the CP group, there was a significant increase in glucose levels and a reduction in triglycerides and VLDL cholesterol concentrations in treated groups (SF and DA) at both time points. On the 14^th^ DPI, the DA group showed a significant reduction in triglycerides and VLDL cholesterol levels, along with a marked elevation in glucose level, compared to the SF group.

The HDL cholesterol level was significantly reduced only in the CP group compared to other experimental groups at both time points. The LDL cholesterol level significantly decreased in the CP group compared to the CN and DA groups on the 14^th^ DPI **(**Table 5**).**

**Table 5:** Glucose and lipid profile of different experimental groups

Parameter Groups		Glucose mg/dl	Triglycerides mg/dl	Total cholesterol mg/dl	HDL cholesterol mg/dl	LDL cholesterol mg/dl	VLDL cholesterol mg/dl
Day zero	CN	148.02 ± 4.70	106.10 ± 2.54	149.37 ± 4.22	66.63 ± 2.81	61.53 ± 1.11	21.22 ± 0.51
	CP	148.59 ± 4.65	102.03 ± 2.85	145.36 ± 4.46	60.56 ± 4.23	64.40 ± 0.39	20.40 ± 0.57
	DA	148.95 ± 3.93	103.25 ± 4.01	148.37 ± 5.67	56.63 ± 4.24	71.09 ± 0.69	20.65 ± 0.80
	SF	147.66 ± 4.26	96.35 ± 5.63	157.90 ± 4.78	69.39 ± 1.97	69.24 ± 1.68	19.27 ± 1.13
10^th^ DPI	CN	143.69 ± 0.55	104.88 ± 3.73	165.66 ± 2.19	74.64 ± 4.84	70.04 ± 2.74	20.98 ± 0.75
	CP	104.76 ± 1.63 ^a^	162.19 ± 1.86 ^a^	163.16 ± 2.71	60.78 ± 2.54 ^a^	69.94 ± 3.67	32.44 ± 0.37 ^a^
	DA	115.84 ± 1.07 ^ab^	120.73 ± 2.82 ^ab^	172.56 ± 0.65	74.75 ± 3.00 ^b^	73.66 ± 1.79	24.15 ± 0.56 ^ab^
	SF	116.28 ± 0.27 ^ab^	128.05 ± 1.41 ^ab^	168.42 ± 1.74	79.14 ± 1.97 ^b^	63.67 ± 0.05	25.61 ± 0.28 ^ab^
14^th^ DPI	CN	147.04 ± 1.48	104.47 ± 2.67	158.60 ± 3.16	68.47 ± 1.85	69.24 ± 2.75	20.89 ± 0.53
	CP	74.45 ± 2.35 ^a^	367.41 ± 3.23 ^a^	166.41 ± 3.16	45.50 ± 1.50 ^a^	47.50 ± 3.96 ^a^	73.42 ± 0.70 ^a^
	DA	141.78 ± 1.44 ^b^	115.86 ± 3.52 ^b^	154.71 ± 2.70	67.77 ± 1.03 ^b^	64.61 ± 3.30 ^b^	22.32 ± 1.62 ^b^
	SF	116.99 ± 2.23 ^abc^	171.95 ± 3.51 ^abc^	160.94 ± 3.61	67.60 ± 2.25 ^b^	58.95 ± 0.65	34.39 ± 0.70 ^abc^

Data are expressed as means ± SE (*n* = 5) with dissimilar superscript letters (significantly differing at *P < 0.05*): (a) significantly different from CN group; (b) significantly different from CP group; (c) significantly different from DA group. CN: negative control group; CP: positive control group (infected); DA: infected and diminazene aceturate-treated group; SF: salvia fraction-treated and infected group. HDL: high-density lipoprotein; LDL: low-density lipoprotein; VLDL: very low-density lipoprotein. DPI: day post-infection

### Brain oxidative stress biomarkers and AChE activity on the 14^th^ DPI

**Fig. 2:**
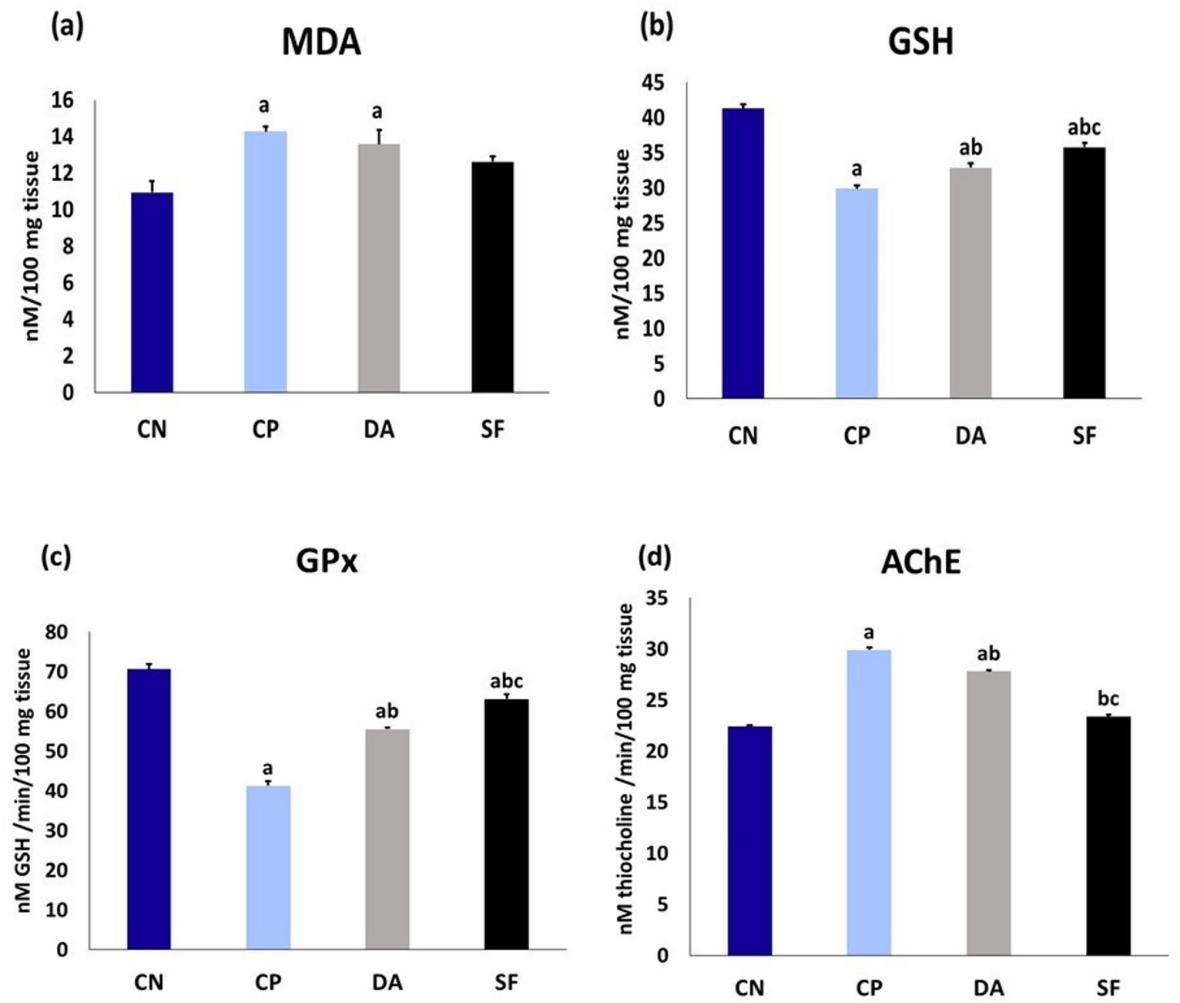
Brain oxidant/ antioxidant biomarkers and AChE activity in different experimental groups on the 14^th^ DPI. Data are expressed as means ± SE (*n* = 5) with dissimilar superscript letters (significantly differing at *P<0.05*): **a**) significantly different from CN group; **b**) significantly different from CP group; **c**) significantly different from DA group. CN: negative control group; CP: positive control group (infected); DA: infected and diminazene aceturate-treated group; SF: salvia fraction-treated and infected group. MDA: malondialdehyde; GSH: reduced glutathione; GPx: glutathione peroxidase; AChE: acetylcholinesterase; DPI: day post-infection

In comparison to the CN group, *T. evansi* infection resulted in a significant decrease in the GSH content and GPx activity in all infected groups. In contrast, the MDA content and AChE activity were markedly elevated only in the CP and DA groups. In both the DA and SF groups, a significant improvement in the GSH content, as well as GPx and AChE activities, was observed when compared with the CP group. The improvement in GPx and AChE activities, along with the increase in GSH content, was more pronounced in the SF group than in the DA group (Fig. 2).

### Gene expression levels of inflammatory cytokines

**Fig. 3:**
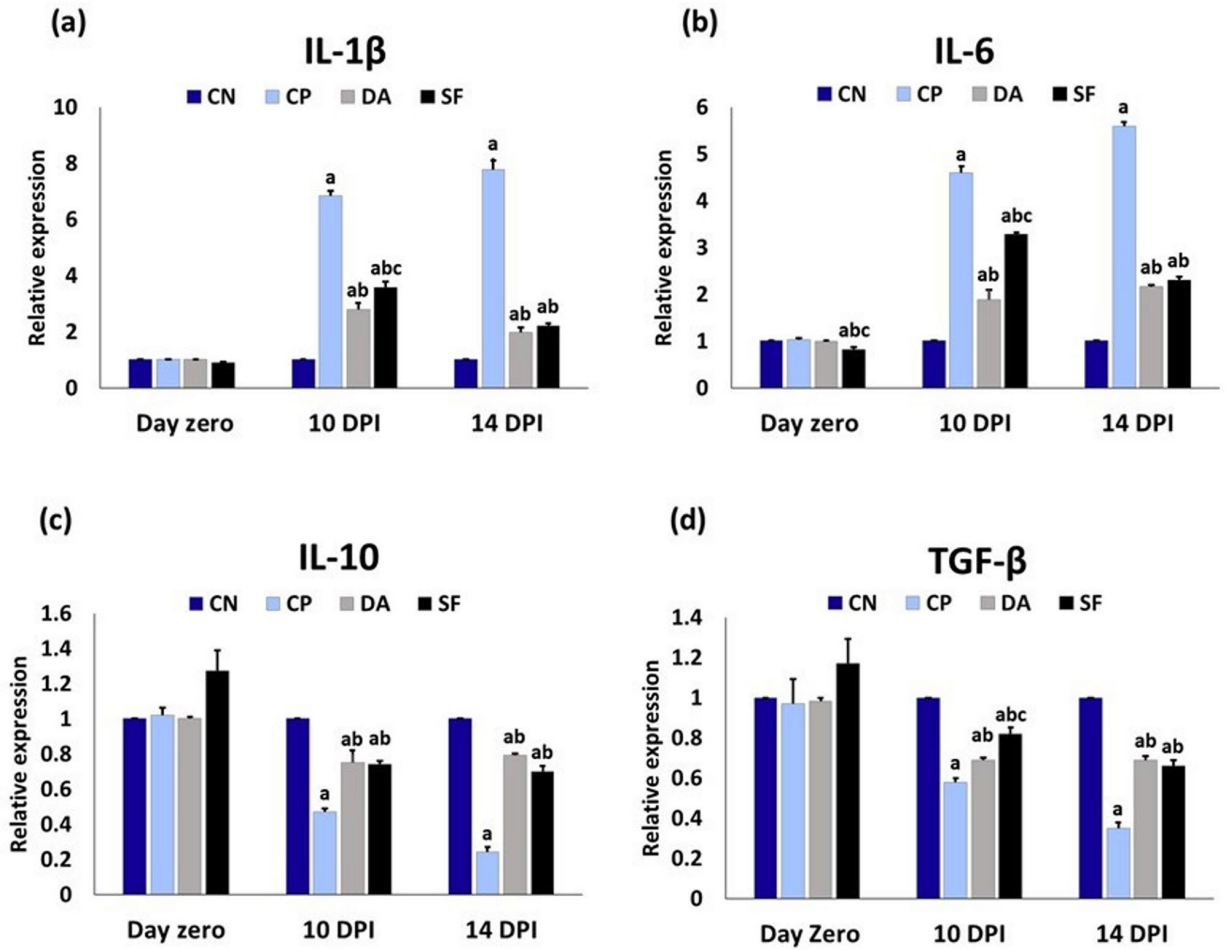
The transcript levels of inflammatory cytokines in the blood of different experimental groups. Data are expressed as means ± SE (*n* = 5) with dissimilar superscript letters (significantly differing at *P < 0.05*): **a**) significantly different from CN group; **b**) significantly different from CP group; **c**) significantly different from DA group. CN: negative control group; CP: positive control group (infected); DA: infected and diminazene aceturate-treated group; SF: salvia fraction-treated and infected group. IL-1β: interleukin-1beta; IL-6: interleukin-6; IL-10: interleukin-10; TGF-β: transforming growth factor beta; DPI: day post-infection

Figure 3 shows that salvia fraction administration resulted in a notable downregulation of IL-6 mRNA level on day zero compared to other experimental groups. On the 10^th^ and 14^th^ DPI, the infection with *T. evansi* induced a significant upregulation of IL-1β and IL-6 mRNA expression levels, whereas mRNA expression levels of TGF-β and IL-10 were downregulated in all infected groups when compared to the CN group.

However, in comparison to the CP group, these responses prominently improved following treatment of infected groups, i.e., the IL-1β and IL-6 expression levels were significantly reduced, and the expression levels of IL-10 and TGF-β were significantly increased in both the DA and SF groups relative to the CP group at the same time points. Diminazene aceturate induced a more pronounced reduction in IL-1β, IL-6, and TGF-β expression levels than the salvia fraction only on the 10^th^ DPI.

### Histopathological findings

**Fig. 4:**
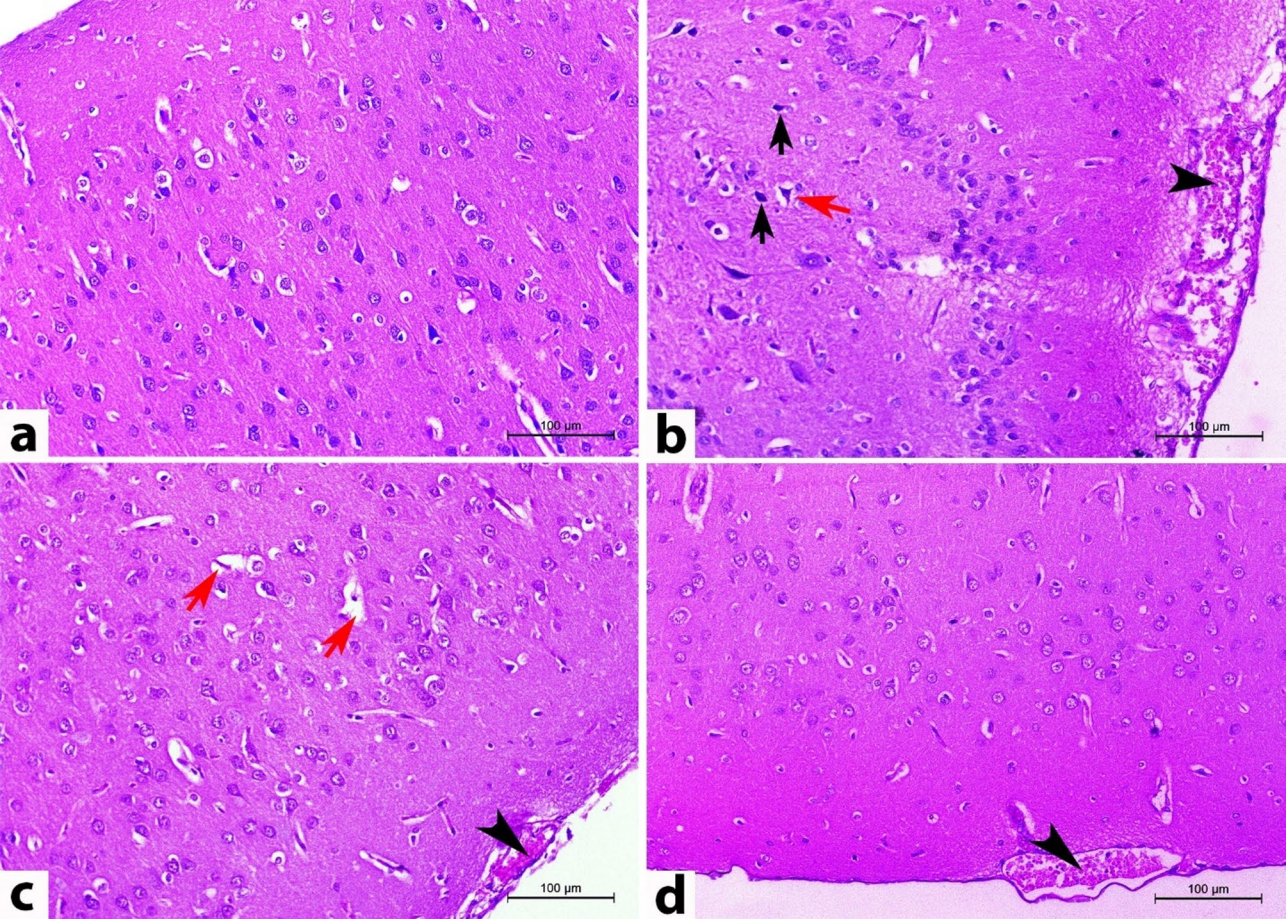
Representative photomicrographs of rat cerebral cortex sections stained with H&E on the 14^th^ DPI (magnification 200X). (**a**) The cerebrum of normal rats revealed the regular arrangement of cerebral cortex layers. (**b**) The cerebrum in the CP group showed disarrangement of cerebral cortical layers, congestion (arrowhead), perineuronal edema (red arrow), and pyknotic nuclei of some neurons (black arrow). (**c**) Cerebral tissues of DA group showed similar cerebral neuropathology but in a lesser degree of severity: congestion (arrowhead) and perivascular edema (red arrow). (**d**) Rats in the SF group had similar cerebral neuropathology to the DA group with more amelioration: congestion (arrowhead)

**Fig. 5:**
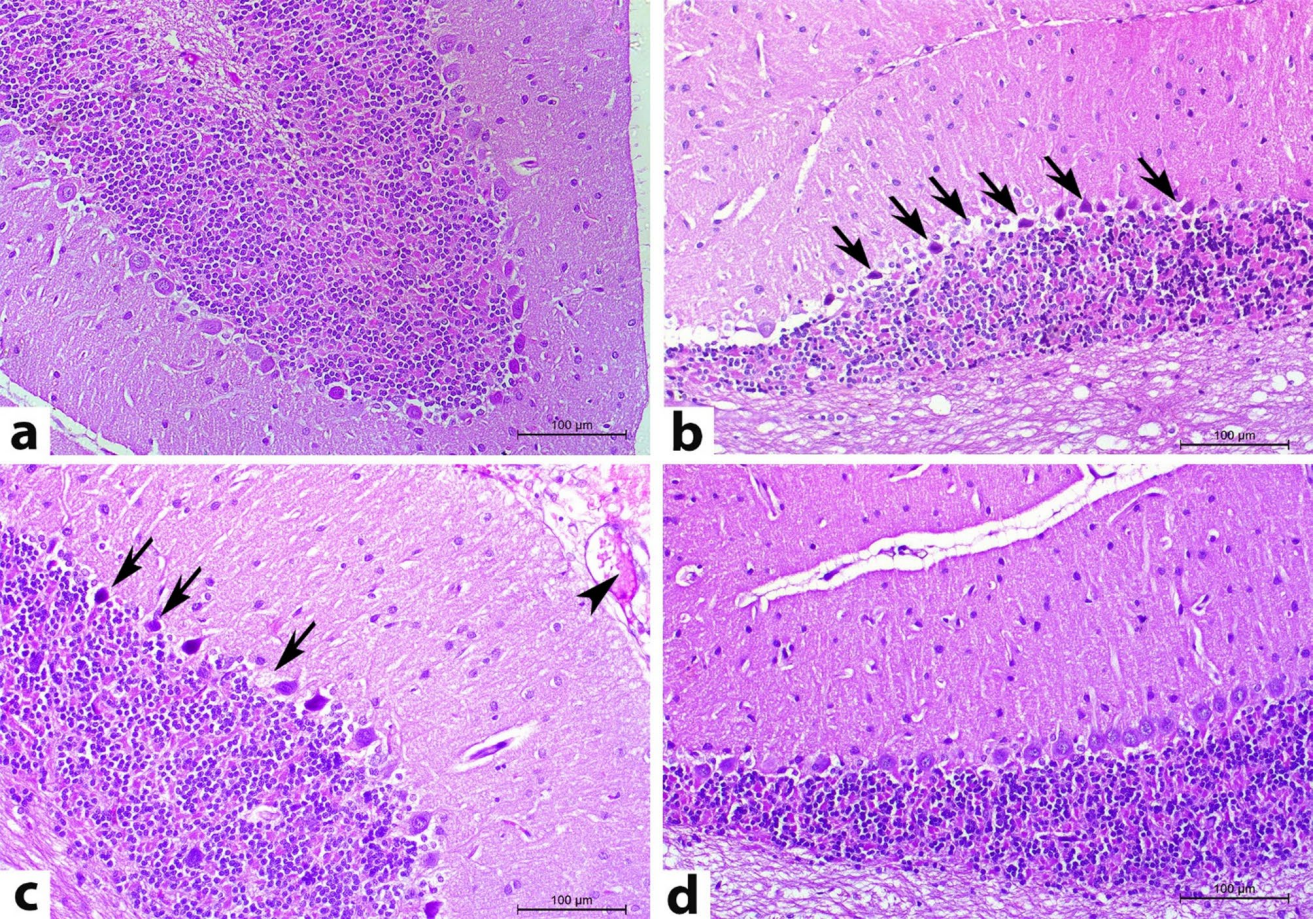
Representative photomicrographs of rat cerebellar cortex sections of rats stained with H&E on the 14^th^ DPI (magnification 200X). (**a**) Normal rats showed a regular arrangement of the cerebellar cortex layer. (**b**) Cerebellar tissues of the CP group exhibited multifocal Purkinje cell necrosis and loss (arrow), congestion, and atrophy of the granular cell layer. (**c**) Rats in the DA group showed the above-described cerebellar lesions but in a lesser degree of severity and distribution: Purkinje cell necrosis (arrow) and congestion (arrowhead). (**d**) The cerebellum of rats in the SF group revealed similar cerebellar neuropathology to the DA group but with more improvement

**Fig. 6:**
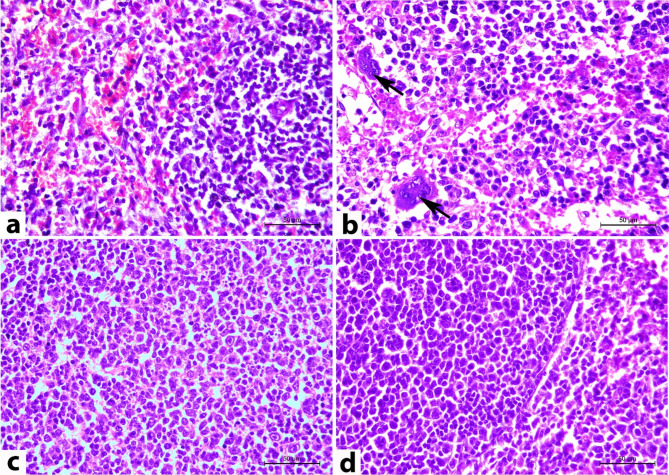
Representative photomicrographs of rat splenic sections stained with H&E on the 14^th^ DPI (magnification 400X). (**a**) Rats in the CN group showed normal histological architecture of the spleen composed of white and red pulps. (**b**) Rats in the CP group revealed proliferation of reticuloendothelial cells and lymphoid cells with many extra-medullary megakaryocytes (arrow). (**c**) Splenic tissue in the DA and (**d**) in the SF groups showed improvement compared to those of the rats in the CP group

The histopathological changes of cerebral, cerebellar, and splenic tissues on the 14^th^ DPI are illustrated in Figs. 4, 5, and 6, respectively.

H&E-stained cerebral sections of normal control rats (CN) revealed the regular arrangement of six layers of the cerebral cortex, named from the outer to the inner as follows: the outer molecular (plexiform) layer (covered with pia matter), the external granular layer, the external pyramidal cell layer, the internal granular layer, the internal pyramidal layer, and finally, the polymorphic cell layer (Fig. 4a). In contrast, most of the cerebral sections from rats inoculated with *T. evansi* (CP) showed a disruption in the organization of the cerebral cortical layers. There was marked congestion of cerebral blood vessels, accompanied by neuronophagia, gliosis, perivascular and perineuronal edema, alongside perivascular cuffing of mononuclear cells. The molecular layer exhibited a considerable number of shrunken and vacuolated neurons. Many neurons showed pyknotic or karyolitic nuclei. Some rats displayed a mild infiltration of lymphocytes into the meninges, with several trypomastigotes detected within the meningeal and cerebral blood vessels (Fig. 4b and Supplementary Fig. 1). On the other hand, rats treated with diminazene aceturate (DA) exhibited the aforementioned cerebral neuropathology, albeit with a reduced degree of severity and extent compared to the CP group, and the parasite was undetectable (Fig. 4c). Salvia fraction-treated rats (SF) showed similar cerebral neuropathology to the DA group but with greater amelioration (Fig. 4d).

H&E-stained cerebellar sections of normal control rats (CN) demonstrated the regular arrangement of three layers of the cerebellar cortex, named from the superficial to the deepest part: the molecular layer (covered with pia matter), the Purkinje cells layer (piriform layer), and the granular cells layer (Fig. 5a). In contrast, most of the cerebellar sections from rats inoculated with *T. evansi* (CP) exhibited multifocal necrosis and depletion of Purkinje cells, alongside congestion and marked atrophy of the granular cell layer, albeit the distribution pattern wasn’t typically observed across all examined cerebellar microscopic fields (Fig. 5b). On the other hand, the rats in the DA group revealed the aforementioned cerebellar neuropathological changes but with diminished severity and extent in comparison to the CP group, and the parasite couldn’t be detected (Fig. 5c). Rats in the SF group had similar cerebellar neuropathology but with marked improvement in comparison to those in the CP and DA groups (Fig. 5d).

Rats in the control group (CN) showed normal histological architecture of the spleen, comprising white and red pulps (Fig. 6a). In contrast, rats inoculated with *T. evansi* (CP) exhibited enlargement of splenic pulps attributed to increased proliferation of reticuloendothelial and lymphoid cells. Concurrently, some rats displayed lymphoid depletion and necrosis in the white pulps, accompanied by numerous apoptotic bodies. Extra-medullary megakaryocytes within red pulps were noted in all spleen sections of both inoculated and non-inoculated rats; however, a significantly higher number was observed in the infected rats compared to their non-infected counterparts. The hemosiderin pigment couldn’t be detected in the splenic tissue sections of either the non-inoculated or inoculated groups. The splenic vasculature showed signs of angiopathy, characterized by endothelial swelling, sloughing, and vacuolar degeneration of the tunica media (Fig. 6b). These histopathological lesions were detected in the spleen of rats belonging to the DA (Fig. 6c) and SF (Fig. 6d) groups, albeit with reduced severity. Rats in the DA group demonstrated marked improvement, while the SF group showed moderate improvement in comparison to the CP group.

## Discussion

Currently, *T. evansi* is a significant challenge in camel-producing countries, leading to substantial economic losses. The biggest obstacle facing *T. evansi* infections is the toxicity and resistance associated with existing chemotherapies [30]. Consequently, natural products and their constituents are promising sources for developing novel medications and alternative therapeutic strategies for trypanosomiasis [15, 31].

Although the salvia fraction administration didn’t induce complete clearance of the parasite from the bloodstream, it significantly reduced parasitemia degree compared to the non-treated and infected group. This reduction may be thanks to diterpenes of the rosmanol and/or rosmaquinone group (43%), phenolic diterpenes (8.3%), and triterpenes (18.3%) in the used fraction. A previous study by Llurba Montesino et al. [13] substantiated the in vitro trypanocidal efficacy of *S. officinalis*, indicating that abietane-type diterpenes of the rosmanol/rosmaquinone group, 12-O-Methylcarnosic acid (phenolic diterpene), and cirsimaritin (flavonoid) isolated from *S. officinalis* tincture had potent in vitro activity against *Trypanosoma brucei rhodesiense*, with the diterpenes of the rosmanol/rosmaquinone group demonstrating the most pronounced activity. Also, da Silva Ferreira et al. [32] stated that ursolic acid, a pentacyclic triterpenoid, displayed trypanocidal efficacy against *Trypanosoma cruzi*. The potential trypanocidal mechanisms of action of these terpenoids include parasite DNA fragmentation, depolarization of the mitochondrial membrane potential of the parasite, and intracellular ATP depletion [33].

Anemia is a cardinal sign of trypanosomiasis [34]. In our study, the reduced RBCs, PCV, and Hb values may be due to hemolytic anemia associated with the *T. evansi* infection. Our findings align with those of Darwish et al. [35] and Razin et al. [36]. The destruction of RBCs might be due to the presence of trypanosomes and their metabolites in the bloodstream, the hydrolysis action of the trypanosomal sialidase enzyme on the RBC membrane, accompanied by the inhibition of erythropoiesis, as well as oxidative damage to the RBCs [36, 37]. The MCV and MCHC values didn’t differ significantly among the experimental groups, characterizing normocytic–normochromic anemia, parallel with Da Silva et al. [38] and Do Carmo et al. [34].

The present study demonstrates that leukocytosis, lymphocytosis, neutrophilia, and monocytosis associated with *T. evansi* infection are consistent with the former report of Do Carmo et al. [34]. It may indicate an immune response against the parasite [35]. Conversely, Razin et al. [36] documented leucopenia associated with *T. evansi* infection in rats.

The parasite clearance from circulation in trypanosomiasis predominantly relies on antibody-mediated phagocytosis, the production of pro-inflammatory cytokines such as TNF-α, IL-1, and IL-6, as well as the release of nitric oxide by classically activated macrophages [5]. Despite the pivotal role of inflammatory cytokines in disease resistance, the massive production of pro-inflammatory cytokines results in an inability to control parasitemia and causes collateral tissue damage [39].

IL-10 plays an essential role in determining the outcome following trypanosome infection. Owing to its anti-inflammatory potential, it modulates the excessive activities of macrophages and T cells, which are responsible for the production of pro-inflammatory cytokines [5].

TGF-β is considered a multifunctional cytokine produced by various tissues. It plays a crucial role as an immunosuppressive agent essential for maintaining immune homeostasis. Generally, it suppresses the activity of immunocompetent cells, i.e., it prevents the differentiation of naïve T-cells into classical effector T-cells (CD4^+^ (helper) and CD8^+^ (cytotoxic) [40].

The reduction in IL-1β and IL-6 levels, coupled with the increase in IL-10 and TGF-β gene expression levels in rats receiving salvia fraction, emphasizes the anti-inflammatory efficacy of *S. officinalis*, as described by previous studies [41, 42].

The remarkable elevation in the expression levels of IL-10 and TGF-β genes in rats treated with salvia fraction may have a role in reducing parasitemia. Onyilagha and Uzonna [5] stated that the elevation in IL-10 and interleukin-4 (IL-4) gene expression levels, alongside a reduction in nitric oxide, is interrelated with protection in cattle infected with *T. congolense*. Moreover, CD8^+^ T cell-deficient mice infected with *T. brucei brucei* exhibited a lower degree of parasitemia when compared to their wild-type counterparts.

Consequently, the improvement of hematological and cytokine gene expression profiles by salvia fraction may be due to its trypanocidal and anti-inflammatory properties, which subsequently reduce parasitemia and the severity of related alterations.

Previous research by Do Carmo et al. [34] reported that rats experimentally infected with *T. evansi* showed hypoglycemia, hypertriglyceridemia, and VLDL cholesterol elevation. These findings are in agreement with the results of the present study. Hypoglycemia is a common laboratory finding in trypanosomiasis, resulting from excessive blood glucose utilization by circulating parasites and liver degeneration hindering the hepatic gluconeogenic pathways [43]. Elevation of triglycerides and VLDL cholesterol associated with the disease is attributable to inhibiting lipoprotein lipase activity via pro-inflammatory cytokines, leading to impaired triglycerides degradation [34, 35].

The decrease in HDL and LDL cholesterols in our study is compatible with the report by Razin et al. [36]; however, the previous report by Do Carmo et al. [34] found that the infection with *T. evansi* induced a non-significant decrease in HDL-cholesterol. These alterations may be a sequel of the parasite’s consumption of host lipids and cholesterol, owing to its inability to synthesize them for growth and multiplication. Moreover, the decrease in HDL cholesterol might be due to the oxidative stress induced by *T. evansi* infection, as the HDL cholesterol has anti-oxidant potential [35].

It was observed that *S. officinalis* terpenoids-rich fraction and diminazene aceturate enhanced blood glucose level and lipid profile. This improvement is possibly a consequence of the decrease in the circulating number of trypanosomes in the bloodstream, besides the anti-inflammatory and antioxidant effects of salvia fraction components.

The involvement of the CNS in the pathogenesis of trypanosomiasis has been discussed by various studies [44, 45]. The AChE is an essential enzyme that hydrolyzes acetylcholine (ACh) neurotransmitter, thereby regulating its level in the synaptic cleft. Consequently, it plays a significant role in mental functions like learning and memory [46]. Our results demonstrated a significant elevation in AChE activity associated with *T. evansi* infection in the CP and DA groups, corroborating the results reported by Baldissera et al. [47]. This increase may be a compensatory mechanism for the elevation of acetylcholine neurotransmitter, as *T. evansi* infection results in a deficit of cerebral Na^+^, K^+^-ATPase activity, which leads to hyperpolarization of the neuronal cell membrane and consequently facilitates the release of more neurotransmitters, including Ach [48]. This alteration in neural activity may occur directly due to the parasitic effects in brain tissue or may arise indirectly as an inflammatory response to the parasite [47], as proved by brain histopathological findings in our study.

The restoration of elevated AChE activity to levels approaching those of the control may result from the acetylcholinesterase inhibitory properties of *S. officinalis* [49]. Conversely, the persistent elevation of AChE activity in the diminazene aceturate group may be due to its inability to cross the blood-brain barrier [50].

Generally, exposure to the pathogen for a long time provokes free radical production and antioxidant depletion, resulting in tissue damage and oxidative stress [36]. In the current study, *T. evansi* infection induced oxidative stress in brain tissue, as evidenced by an elevation in MDA concentration and a reduction in GSH content and GPx activity. These findings are consistent with a previous study conducted by Dkhil et al. [45], who found an increase in MDA and a decrease in GSH concentrations in the brains of mice experimentally infected with *T. evansi* on the 4^th^ DPI. While in our study, it increased on the 14^th^ DPI, as the elevation in the brain MDA in both studies was usually associated with exposure to the protozoan, regardless of the time point.

The treatment with salvia fraction markedly improved the brain’s oxidative status as the antioxidant properties of *S. officinalis* are well-known [42, 51]. This antioxidant efficacy may have a role in the pathophysiology of anemia, i.e., reducing oxidative damage to red blood cells.

Despite the antioxidant potential of *S. officinalis*, the treatment with salvia fraction couldn’t restore the GPx activity to the level of non-infected rats. It may be due to oxidative stress associated with the persistent parasitemia.

The concomitant histopathological alterations in the brain and spleen with trypanosomiasis have been demonstrated by several studies [35, 36, 45]. These changes are usually a result of intravascular parasite multiplication, alongside oxygen consumption, leading to hypoxia and consequent degenerative changes. Also, the oxidative stress and liberated toxins from *T. evansi* may have a role [15, 52].

The megakaryocytes, as extra-medullary hematopoiesis (EMH) in splenic tissue, are a normal feature in rodents [53]. The prominent increase in splenic megakaryocytes observed in this study may represent a compensatory response to anemia induced by *Trypanosoma* infection [54]. Additionally, the vascular angiopathy associated with the disease may further contribute to this finding. In this study, the proliferation of reticuloendothelial cells could be a compensatory mechanism aimed at clearing erythrocytes coated with the parasite antigens [52]. The cellular apoptosis observed with *T. evansi* infection may be induced by toxic products of trypanosomes. According to Ramadan et al. [55], mice experimentally infected with *T. evansi* exhibited a significant upregulation of caspase-3 in their splenic tissues, indicating its role in apoptosis.

The administration of salvia fraction mitigated the histopathological picture, which may be due to its antioxidant, anti-inflammatory and antitrypanosomal potentials. The neuroprotective effect of *S. officinalis* was also reported by Ayoub et al. [51].

## Conclusion

Based on the present findings, the oral administration of *S. officinalis* terpenoids-rich fraction exhibited in vivo antitrypanosomal efficacy by reducing the level of parasitemia. Additionally, it alleviated the degree of anemia, leucocytic response, hypoglycemia, alterations in lipid profile, brain oxidative stress, and histopathological changes associated with *T. evansi* infection. Furthermore, it modulated the inflammatory cytokines after *T. evansi* infection. Consequently, this study suggests that these fraction components could be of value for novel therapeutic agents for the treatments of trypanosomiasis.

## Electronic Supplementary Material

Below is the link to the electronic supplementary material.

Supplementary Figure 1

## Data Availability

The research data used to support the findings of this study are included within the article.
