# Supplementary material for: Antitrypanosomal potential of Salvia officinalis terpenoids-rich fraction in Trypanosoma evansi-infected rat model
Source: BMC Vet Res. 2025 Jun 27;21:410. doi: 10.1186/s12917-025-04861-2 (PMC12203732; doi:10.1186/s12917-025-04861-2)
Supplement: Supplementary file 1 — Supplementary Figure 1 [file 12917_2025_4861_MOESM1_ESM.docx]

**
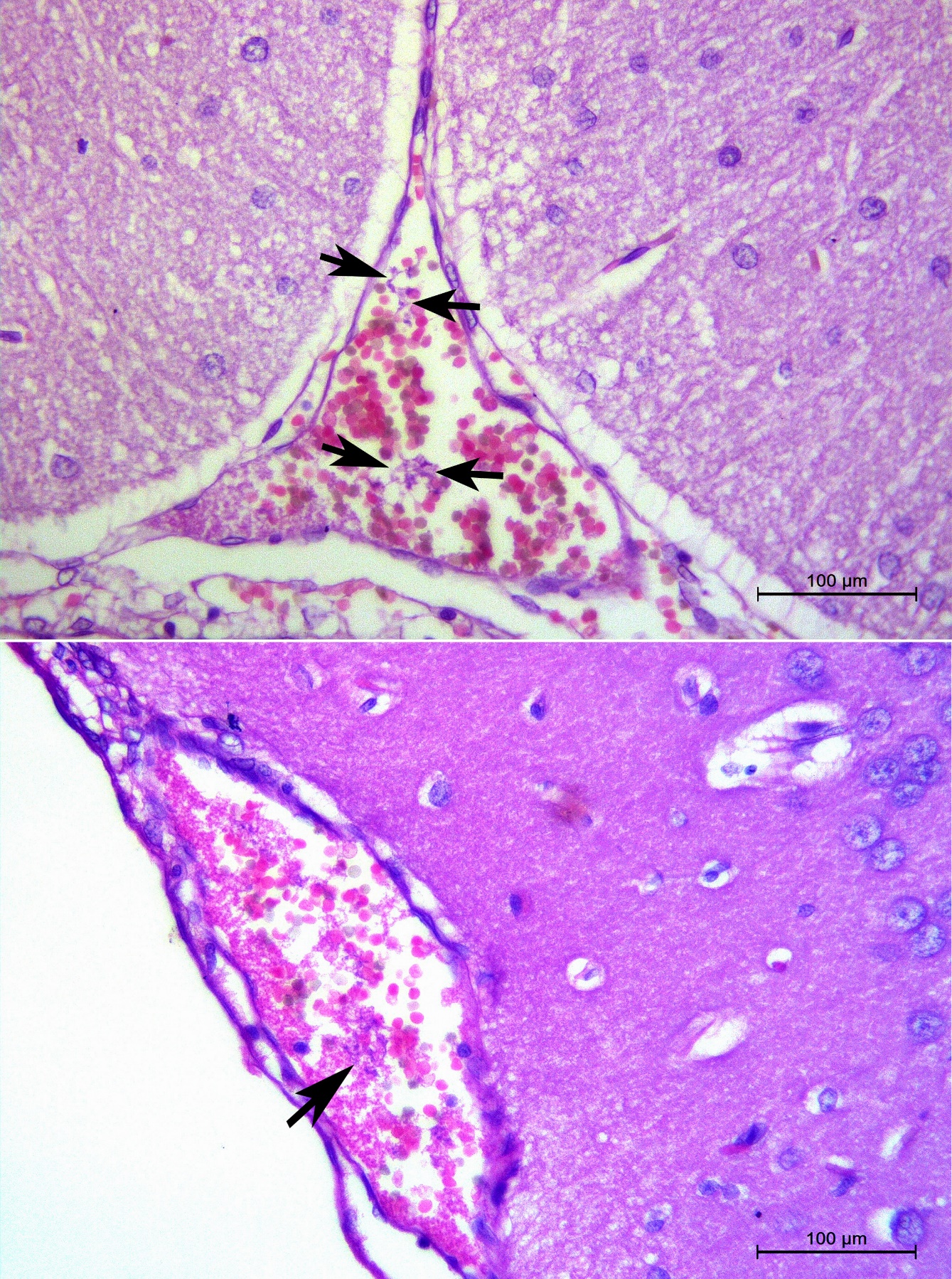
 Supplementary Fig.1** Trypomastigotes within the meningeal and cerebral blood vessels **(black arrow)**.
